# Supplementary material for: The Phosin PptA Plays a Negative Role in the Regulation of Antibiotic Production in Streptomyces lividans
Source: Antibiotics (Basel). 2021 Mar 20;10(3):325. doi: 10.3390/antibiotics10030325 (PMC8003754; doi:10.3390/antibiotics10030325)
Supplement: Supplementary file 1 [file antibiotics-10-00325-s001.pdf]

Table 1 Bacterial strains used in this study

Strains	Description	Source or reference
Escherichia coli		
DH5α	General cloning host strain	Promega
DY330	Strain used for PCR-targeted mutagenesis	Yu *et al.* (2000)
S17.1	Strain used for conjugation from *E. coli* to *S. lividans*	Simon *et al.* (1983)
Streptomyces coelicolor		
M145	$SCP1^{-}$, $SCP2^{-}$	Hopwood *et al.* (1983)
Streptomyces lividans		
TK24	$SLP2^{-}$, $SLP3^{-}$, *strR*	Hopwood *et al.* (1983)
Δ ppk (SLI_4384)	TK24 with *ppk*::Ω*hyg*	Chouayekh *et al.* (2002)
Δ phoP (SLI_4466)	TK24 with *phoP*::Ω*aac*	Ghorbel *et al.* (2006a)
Δ SLI_4382	TK24 with *SLI_4382*::*att3-aac*	This work
Δ pptA (SLI_4383)	TK24 with *SLI_4383*::*att3-aac*	This work
Δ $pptA^{IFD}$	TK24 with *SLI_4383*::*att3*	This work
Δ $pptA^{IFD}$ -C(-)	Δ$pptA^{IFD}$ with pSET152	This work
Δ $pptA^{IFD}$ -C(+)	Δ$pptA^{IFD}$ with pMES18, complementation strain of Δ$pptA^{IFD}$	This work

IFD stands for In Frame Deletion

Table 2 Plasmids used in this study

Plasmids/Cosmids	Description	Source or reference
Construction of mutant strains		
StD84	Cosmid from the S. *coelicolor* genome library containing *SLI_4383* and *SLI_4382*	Bentley *et al.* (2002)
pGEM-T Easy	*E. coli* vector for cloning of PCR DNA fragment, Amp^R	Promega
pOSV236	*E.coli-Streptomyces* shuttle conjugative vector, Amp^R, Tsr^R, Pur^R, containing p*trc-xis-int*, used for apramycin cassette excision	Nguyen *et al.* (2010)
pOSV234	*E. coli-Streptomyces* shuttle vector, source of the *aac* cassette, Amp^R, Apr^R	Karray (2005)
pSET152	*E.coli-Streptomyces* shuttle conjugative vector, replicative in *E. coli*, Apr^R, integrative in *Streptomyces*, used for genetic complementation in *Streptomyces*	Bierman *et al.* (1992)
pMES *pptA*::*aac*	*pptA*::*att3aac* in StD84, Amp^R, Apr^R	This work
pMES *SLI_4382*::*aac*	*SLI_4382* ::*att3aac* in StD84, Amp^R, Apr^R	This work
pMES17	1.5-kpb fragment containing *pptA* and its promoter region in pGEM-T Easy, Amp^R	This work
pMES18	1.5-kpb fragment containing *pptA* and its promoter region in pSET152, AprR	This work

Amp^R; ampicillin resistant, Apr^R; apramycin resistant, Pur^R; puromycin resistant, Tsr^R; thiostrepton resistant

Table 3 Oligonucleotides used in this study

Purpose	Primer Pairs	5' → 3' sequence
**Gene disruption**		
pptA (SLI_4383)	NS1	CGCCCCGCACGCCGGCACCGAAGCGGGCACCGA
		CGCCCTGATCGCGCGCGCTTCGTTCGGGACGAA
	NS2	ACGCCTCCCGTCGTCCTGCGGCCGTTCCTGCGA
		CCGTTCCATCTGCCTCTTCGTCCCGAAGCAACT
SLI_4382	NS3	TCGCAGGAACGGCCGCAGGACGACGGGAGGCGT
		ACGCACCATCGCGCGCGCTTCGTTCGGGACGAA
	NS4	GGGGTGAGTGCGCGGGGGTGAGTGCTCAGGTCT
		GGGTGTTATCTGCCTCTTCGTCCCGAAGCAACT
**Amplification of *pptA* (*SLI_4383)***	ED73	CTCGAGAACGGCATGTCCGA
	EC74	TGCGGCCGTTCCTACGCCAG
**Southern Blot verification of the mutants**	NS-F	TCTTGACGTCGACGGTCTTGATGC
	NS-R	ACCGGACGTCCGGCGACTCCCCCA
**Primers for 5'-RACE PCR**		
	SLI_4382 -GSP1	TAGCCCGTCTCCTCCGCGAC
SLI_4382	*SLI_4382* -GSP2	CCTTCGGGTGCGACCAGTCG
	SLI_4382 -GSP3	GGACGCCACAGTACGCAGCC
	pptA-GSP1	GCTCCAGACGGGACGCGTAC
pptA (SLI_4383)	*pptA*-GSP2	GCGGCTGGAAGGTGTGCAGG
	pptA-GSP3	GTTCGCCGAGGGCACCGTTC
**Primers for qRT-PCR**		
Reference genes		
SLI_6088 (hrdB)	F	AAGGAAGACGGCGAGCTTCT
	R	GCACCGGGATACGGATGGTG
SLI_2451 (glk)	F	CAGCGCTCCACGGTCTACTT
	R	GTGATGCAGATGACGTTGC
SLI_4040 (aspS)	F	CTGCTGATGATCTCGGGCTT
	R	CGAGCTGGTAGAACTCGCC
SLI_4129 (gyrA)	F	GGCGACTCCTCCATCTACGA
	R	GACCATCTCCATCGACAGC
SLI_4131 (gyrB)	F	TCGAGACCACCGACTACTCCTT
	R	TCTTGACCTCGTGCTTCTCG
SLI_4926 (rpoB)	F	CTTCGAGCCTCCCAAGAACA
	R	TTGGTCATGAGCGGGAAGT
Target genes		
Ppk (SLI_4384)	F (A)	AGTCCGCCAACATCAAGTGG
	R (B)	GCTTGCAGTGGGTCTTCAGG
ppk-pptA junction	F (C)	CTGCGCAACATCCAGGAGAT
	R (D)	GGGGTTTCCGGTGGAAAGT
pptA (SLI_4383)	F (E)	GTACGCGTCCCGTCTGGAG
	R (F)	GCCCACCGTGAGGTTGC
pptA -SLI_4382 junction	F (G)	CAGCGGCATGAGGTGGAG
	R (H)	TCGTCTGCGGGGCTCA
SLI_4382	F (I)	CCCGAAGTACGACGACTGGT
	R (J)	GAGGTAGCGCACGGTGGTC
**Primers for RT-PCR**		
pptA (SLI_4383)	F (K)	TCTCCGGTTCGACGGCGTTC
	R (L)	TGGCCAGTAAGGCGACCTTG
